# Chemical and microbiological assessment of drinking water quality

**DOI:** 10.4314/ahs.v22i4.70

**Published:** 2022-12

**Authors:** Pınar Mursaloğlu Kaynar, Figen Demli, Günnur Orhan, Hüseyin İlter

**Affiliations:** 1 Hitit University, Alaca Avni Çelik Vocational School, Çorum; 2 Ministry of Health, General Directorate of Public Health, Ankara; 3 Gazi University, Health Services Vocational School, Ankara; 4 Ministry of Health, Ankara Provincial Health Directorate, Ankara

**Keywords:** Drinking water, analysis, chemical, microbiological, quality

## Abstract

**Background:**

Access to an adequate amount of water is restricted because of the increase in the use of natural resources, which is caused by the rapid growing in world population and the climate change that global warming brings, and the development in the industry. Investigating the causes of water pollution, creating solutions for the problem, taking the control of the pollution, and maintaining monitorability are necessary.

**Objectives:**

This study was carried out in order to determine microbiological and chemical characteristics of drinking water and their compatibility for human consumption with the aim of providing safety of drinking waters.

**Methods:**

Thirty-four drinking water samples obtained from different sampling points in Ankara, Turkey, in 2019 were subjected to microbiological analysis and chemical analysis in terms of anions (bromide-Br-, chloride-Cl-, fluoride-F-, nitrate-NO3-, nitrite-NO2-, sulfate-SO4-2) by ion chromatography. Microbiological analyses were applied according to the international standards.

**Results:**

None of the samples contained coliform bacteria, *Escherichia coli* and intestinal enterococci. It was established that concentrations of the specified anions in tested waters were within the acceptable levels of with the Council Directive 98/83/EC.

**Conclusions:**

The determined chemical and microbiological qualities of these samples are suitable for drinking, and do not pose any threats to public health.

## Introduction

Drinking water is used for drinking and other domestic purposes, such as cooking, washing-up, bathing, and showering. The quality of water, essential for a healthy life, is extremely important. For public health, drinking water must not constitute a physical, microbiological, and chemical risk. These hazards must also not pose a direct or indirect health risk. Drinking water of unreliable microbiological quality can result in gastrointestinal diseases and is also responsible for deaths^1^,^2^. Various studies have therefore investigated the quality of drinking water, in different parts of the world, for improving drinking water safety and maintaining public health^3^–^5^. Chemical characterization in drinking water may increase the risk of cancer, hypertension, kidney disease etc^7^–^8^. It is obligated that drinking water is wholesome and clean to protect human health from the adverse effects of any contamination. The drinking water legislation is to object the quality and safety of water intended for human consumption^9^. The purpose of this study was to determine microbiological and chemical characteristics in drinking waters, and their compatibility for human in Ankara in Turkey.

## Materials And Method

### Sampling

In this research, a total of 34 drinking water samples from determined different sampling points in Ankara, Turkey, in Autumn in 2019. 1-liter of plastic bottles were used and samples were collected according to EN ISO 19458^10^, EN ISO 5667-1^11^, EN ISO 5667-1/AC^12^, EN ISO 5667-3^13^. Samples were numbered and coded based on the order in which they were obtained. The samples were transferred into the laboratory as soon as possible during the same day after finishing collection. They were subjected to microbiological and chemical analysis.

### Determination of coliform bacteria and *E. coli* in the water samples

EN ISO 9308-1^14^ and EN ISO 9308-1/A1^15^ were used for the enumeration of coliform bacteria and *E. coli*. For the enumeration of these bacteria, 100 mL of water samples were filtered with 0.45 µm diameter sterile membrane filters (Sartorius) and these filters were placed on Ready Plate Chromocult Coliform Agar (Merck). The media were incubated at 36±2°C for 21 to 24 h. After the incubation, all colonies giving a positive β-D-galactosidase reaction (pink to red) as presumptive coliform bacteria that were not *E. coli* were counted. Also, all colonies giving dark-blue to violet color (a positive β-D-galactosidase and a β-D-glucuronidase reaction) such as *E. coli* were counted. To confirm the presumptive coliform bacteria, oxidase test strips (Merck) were used according to EN ISO 8199^16^.

### Determination of intestinal enterococci in the water samples

EN ISO 7899-2^17^ was used for the enumeration of intestinal enterococci. 100 mL of water samples were filtered with 0.45 µm diameter sterile membrane filters (Sartorius). These filters were placed on Slanetz and Bartley Agar (Oxoid) and were incubated at 36±2 °C for 44±4 h. After the incubation, if typical colonies, which had shown a red, maroon, or pink colour, either in the center or throughout, were observed on membrane filtres, these filtres were transferred Bile-Aesculin-Azide Agar (Sigma) and incubated at 44±0.5°C for 2 h. To confirm the presumptive intestinal enterococci, all typical colonies showing a tan to black color in the surrounding medium were counted.

### Determination of anions (bromide, chloride, fluoride, nitrate, nitrite, and sulfate)

EN ISO 10304-1^18^ and EN ISO 10304-1/AC^19^ for the analysis of the concentration of anions (bromide, chloride, fluoride, nitrate, nitrite, and sulfate) by ion chromatography. The Dionex 5000+ IC System (Thermo Scientific, USA) (molecular conductivity detector, suppressor: ASRD-4 mm/anion, current: 112 mA, column temperature: 30°C, cell temperature: 35°C, flow rate: 1 mL/min, injection volume: 100 µL) was used. Thermo Scientific Dionex IonPac AS19 (4 mm x 250 mm) analytical column and its respective guard column, the Dioex IonPac AG19 (4 mm x 50 mm), were used for the separation of bromide, chloride, fluoride, nitrate, nitrite, sulfate. The mobile phase was 12–45 mM potassium hydroxide (KOH). All solutions were prepared in ultrapure water. Commercially available 1000 mg/L certified solutions of bromide, chloride, fluoride, nitrate, nitrite, and sulfate (Merck) were used to prepare working standard solutions. Certified solutions used to prepare the quality control and the working standards were different.

## Results

At present, the increase in chemical and microbiological pollutions is seen in the waters that are accessed, and they pose threats to public health and the environment. In this study, thirty-four drinking water samples were subjected to microbiological analysis (coliform bacteria, *Escherichia coli*, intestinal enterococci) and chemical analysis in terms of anions (bromide-Br-, chloride-Cl-, fluoride-F-, nitrate-NO_3_-, nitrite-NO_2_-, sulfate-SO_4_^-2^) by ion chromatography. Microbiological analyses were applied according to international standard methods. No growth of these bacteria colonies was observed on the medium. The numbers of these bacteria, which are specified in the Council Directive 98/83/EC^9^ on the quality of water intended for human consumption into the national system for use, colony-forming units per 100 mL water are 0. As results of in this study, it was determined that concentrations of anions in the analysed water samples. These concentrations were compared to the set limits according to the Council Directive 98/83/EC^9^ and found within the acceptable levels of the Directive^9^. Both parameters are expressed in Table 1. Additionally, with the evaluation of the data that were collected, no correlation between microbiological and chemical results is found.

**Table 1:** Anion concentration level (mg·L^-1^) measured in the drinking water samples, compared to the Council Directive 98/83/EC^9^

Water Sample	Chloride	Fluoride	Nitrate	Nitrite	Sulfate
S1	12.60	0.45	11.49	0.05	35.60
S2	8.77	0.86	8.75	0.05	28.20
S3	7.56	0.20	3.92	0.4	27.50
S4	7.49	0.20	3.28	0.05	26.90
S5	7.71	0.28	4.40	0.05	27.30
S6	12.60	0.20	15.99	0.05	38.20
S7	11.90	0.22	15.62	0.05	35.70
S8	9.75	0.19	0.68	0,05	26.40
S9	7.54	0.20	1.36	0,05	28.60
S10	17.80	0.61	16.88	0.05	38.90
S11	11.80	0.49	9.49	0.05	32.60
S12	14.60	0.55	9.21	0.05	33.80
S13	6.99	0.05	1.46	0.05	26.30
S14	6.86	0.04	1.56	0.05	25.60
S15	7.88	0.20	2.09	0.05	28.50
S16	7.08	0.09	1.55	0.05	25.40
S17	7.54	0.14	1.67	0.05	26.70
S18	7.06	0.10	1.07	0.05	27.50
S19	7.15	0.21	2.78	0.05	29.30
S20	7.79	0.20	0.90	0.4	28.60
S21	7.82	0.20	1.26	0.4	28.20
S22	7.74	0.12	1.22	0.4	27.60
S23	7.77	0.20	1.12	0.4	27.55
S24	7.72	0.20	1.30	0.4	28.50
S25	7.67	0.18	12.03	0.05	23.90
S26	7.02	0.05	1.82	0.05	22.70
S27	7.06	0.16	3.56	0.05	28.50
S28	6.93	0.43	4.87	0.05	28.40
S29	7.71	0.26	15.30	0.4	35.50
S30	7.68	0.25	16.42	0.4	32.70
S31	7.04	0.20	9.54	0.05	27.50
S32	7.22	0.15	1.65	0.05	24.10
S33	6.84	0.12	0.90	0.4	23.40
S34	6.92	0.20	9.49	0.4	36.50

Limit Values in Regulatory	250	1.5	50	0.5	250

## Discussion

The quality of drinking water has great importance for human health. Microbiological and chemical characteristics of drinking water are crucial factors that greatly affect water quality. In this study, thirty-four drinking water samples were analysed for microbiological analysis. Conventional standard methods were used for coliform bacteria, *E. coli*, and intestinal enterococci. It was observed that these bacteria colonies (0 cfu/100 mL) had no growth on the medium. Therefore, the drinking water samples in this study complied with the Council Directive 98/83/EC^9^. The microbiological quality of drinking water from different countries has also been evaluated in various studies, which have concluded that bacteria may cause diseases in humans^20^–^22^. The fecal indicator bacteria included *E. coli* and *Enterococcus* were evaluated in drinking water samples and emphasized on required to the provision of safely managed water to improve water quality^23^, ^24^. In this study, drinking water samples were also measured for the concentration of anions, using ion chromatography. The results obtained in this research were compared to the set limits according to the Council Directive 98/83/EC9 (Table 1). Gara et al.^25^ found different results for 21 water-quality parameters of 18 African countries and compared of WHO, EU, US, and China.

Because levels of nitrate and nitrite, which are increased in drinking water can affect human health and aquatic organisms, the nitrite and nitrate levels in the drinking water have been investigated 26, 27. Nitrate and nitrite in drinking water samples were measured in this research. In comparison, to the Council Directive 98/83/EC9 permissible levels for nitrate and nitrite (mg/L), all of the samples exhibited levels below the set standard limits. Fluoride in drinking water is important for dental care, but the level exceeded may cause bone disease and teeth problems. Fluoride levels measured in all the drinking water samples were below the permissible levels (Table 1). Many researchers investigated fluoride which is an occurring health hazard in drinking-water resources^28^,^29^. Chloride and sulphate levels exceeded can change the taste of water and cause gastrointestinal problems, respectively^26^,^30^. In this study, the chloride concentrations of the water samples were between 6.84–17.80 mgL−1. No all the chloride and sulphate levels measured in all the drinking water samples were below the permissible levels for the Council Directive 98/83/EC9 and also the samples' set limit is provided for the bromide from this Directive. Bromide was not detected in all the drinking water samples. D'Alessandro et al.^31^ reported that drinking water samples were analysed for bromide, and the level of bromide exceeded 1 mg/L in about 3% of samples. As a result of this study, it was observed that fluoride (0.04–0.86 mgL−1), nitrate (0.68–16.88 mgL−1), nitrite (0.05–0.4 mgL−1), and sulphate (22.70–38.90 mgL−1), found at different levels (Table 1). Due to microbiologically and chemical hazardous, various studies have investigated the water safety different city in Turkey^32^–^34^.

In conclusion, drinking water samples that were examined could not generally be found microbial and chemical risk which public health may be affected. Therefore, these drinking water samples are found to have good quality. It has also seemed that continuous monitoring of water quality and obeying regulations are required to ensure that the water quality meets the set standards.
